# Family doctors on the front line

**DOI:** 10.4102/safp.v62i1.5138

**Published:** 2020-05-19

**Authors:** Bob Mash

**Affiliations:** 1 Division of Family Medicine and Primary Care, Faculty of Medicine and Health Sciences, University of Stellenbosch, Cape Town, South Africa

**FIGURE 1:**
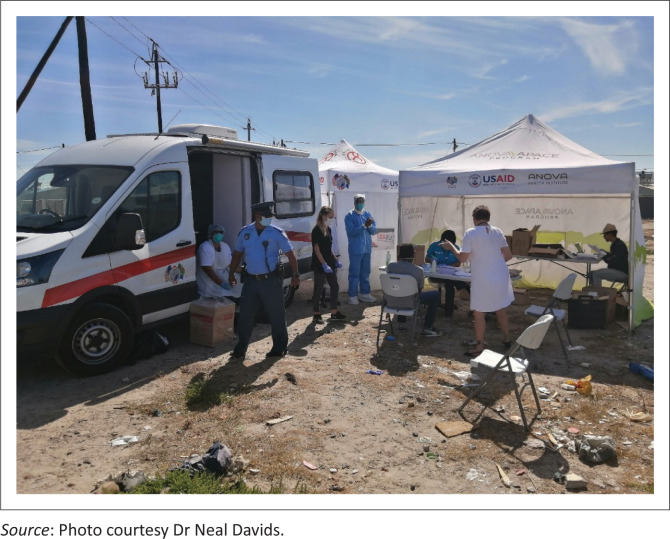
Family doctors assist with community screening and testing in Cape Town.

World Family Doctor Day on 19 May has the theme ‘family doctors on the front line’.^1^ As I write this editorial we have just come out of full lockdown in Cape Town, which now has the most cases of COVID-19 in the country. We are expecting the number of cases to peak during the next few weeks, and when you read this the surge may have already happened. The future is uncertain and family doctors are on the front line.(fig 1)

In Cape Town, family physicians are not only on the front line in terms of primary and district hospital care, but have been instrumental in providing leadership and helping the health services to reorganise and decongest. The primary healthcare preparedness plan for the Western Cape was drafted by a family physician, the community screening and testing has been led by a family physician, the guidance for community health workers was also drafted by a family physician. Family physicians have been engaging in case investigation and contact tracing. Family physicians have also been leading their health centres and district hospitals to reorganise for the expected onslaught.

It has been encouraging to see so many people collaborate and volunteer their assistance. Private general practitioners have been offering to help the public sector. Public health and family medicine specialists have worked together in a community orientated primary care (COPC) approach. Indeed, the foundation that was laid in Cape Town for COPC over the last few years has proved fundamental to a rapid community-based response. For example, community health workers in every poor and vulnerable community were rapidly able to reorganise and assist with home delivery of medication as well as community screening and testing. Professional volunteers have staffed the phones for case investigation, and medical students have assisted with contact tracing. Many non-professional people from faith-based and other organisations also volunteered their assistance.

**FIGURE 2:**
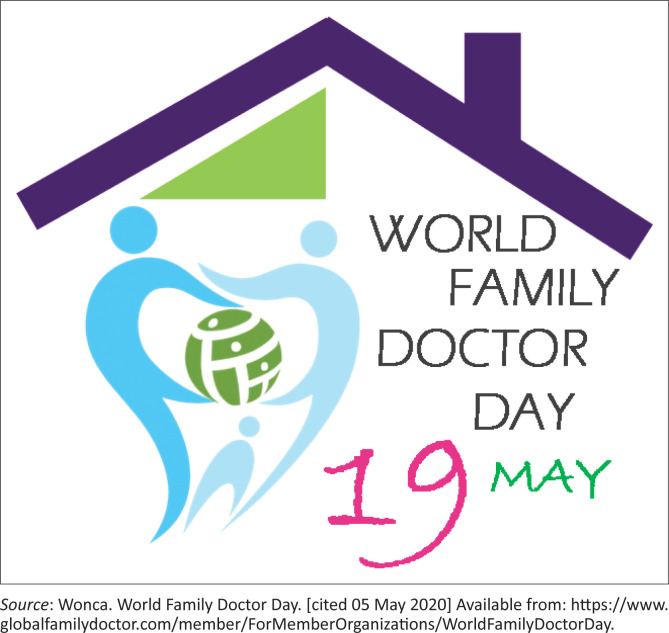
Wonca logo for World Family Doctor Day.

Containment and suppression of the epidemic relies on strong primary healthcare and this relies on strong teams of nurse practitioners, family doctors, professional nurses, community health workers and many others. Most people infected with COVID-19 will be managed in primary care. Leadership from family physicians has been important across all of their roles: clinical care, consultancy to the team, training of staff, building capacity to tackle the epidemic, introducing new guidelines and supporting COPC.(fig 2)

The COVID-19 epidemic has made visible the importance of COPC as well as the value of having a family physician in the team. A recent analysis of the Health Professions Council of South Africa database, however, shows that we only have 0.16 family physicians per 10 000 population. This varies from 0.6 in the public sector to 6.8 per 10 000 in the private sector, and from 0.3 per 10 000 in the Western Cape to 0.05 per 10 000 in Limpopo. Although there is no agreed international benchmark, a minimum of 3.0 per 10 000 has been suggested and most high-income countries have between 4.0 and 12.0 per 10 000.^1^

The South African Academy of Family Physicians has a minimum and short-term goal of having a family physician at every district hospital, community health centre or sub-district in the country.^2^ This would mean a minimum of 680 family physicians in the public sector. In order to reach this target in the next 10 years, the nine training programmes will need to double or even triple their output. This will require a commitment from government and their new human resources for health policy to establish more registrar posts and more family physician posts. We have recently seen the government import 100 family physicians from Cuba to assist with the crisis and we call on them to maintain this level of commitment to our own family physicians.^3^

As we look to the future and the possibility of national health insurance, we also see the need for primary care facilities to include a family doctor, in order to be easily accredited as able to provide the full range of services at the required level of performance and quality. Such family doctors should have postgraduate training in family medicine. Currently such training is available as a two-year diploma or a four-year fellowship.

The South African Academy of Family Physicians will continue to advocate for family doctors and support the discipline through its engagement with stakeholders, publications, conferences, journal research, academic coordination and continuing professional development.

So as we celebrate World Family Doctor Day we acknowledge the importance of a strong COPC and the vital role played by family.
